# Hospital and Institutional News

**Published:** 1920-02-14

**Authors:** 


### HOSPITAL AND INSTITUTIONAL NEWS.

### THE PRINCE OF WALES AT GUY'S.

The Prince of Wales, who is the newly-elected President of Guy's Hospital, paid a visit to that institution on February 5 at 2 p.m. in order to preside over a Court of Governors called to consider the matter of a special appeal for funds, and he later made an inspection of the hospital premises, including several of the wards. As His Royal Highness reached the hospital, attended by the Hon. Sir Sidney Greville, a crowd assembled at the St. Thomas's Street entrance gave the Royal visitor many hearty cheers. The Prince was received by Mr. Cosmo Bonsor, the retiring President, by the governors, the principal members of the medical and surgical staffs, and the matron. After brief proceedings in the board-room, at which we understand that His Royal Highness gave his full approval to the principle of the new appeal, although details of the scheme are still incomplete (a special committee has been appointed to make all necessary arrangements), a short tour of the hospital was undertaken. In thus accepting the Presidency the Prince of Wales is following the example set by the late King Edward, who some years ago became the first Royal President. The subsequent inspection included the hospital chapel, some of the wards, and the park. Turning first into Astley-Cooper Ward, the Prince passed through the colonnade, and received from a crowd of students assembled there a right royal welcome. In the wards His Royal Highness visited practically every patient whose bed he passed, displaying the deepest understanding of their troubles and prospects in convalescence, and exhibiting particular interest in those who had served with the fighting forces. If space would allow, delightful examples of this Royal sympathy might be given, but suffice it to say that the Prince yet again demonstrated that great appreciation, kindness, and consideration for which he is so justly famous. The younger patients came in for especial notice, and will well remember their Royal visitor's charm and words of comfort. Considerable attention was given to the electro-therapeutic department, with its many modern improvements, and then, after expressing his general approval of the many organisations which he saw, the Prince, after a full hour's stay, left the quadrangle, cheered to the echo by the assembled groups of students and the Borough crowd outside the gates.

### PRINCESS MARY AND A LONDON HOSPITAL.

On February 26 Princess Mary will receive purses at the Mansion House on behalf of the London Fever Hospital, Islington, when a reception will be held at which the Lord Mayor, Sir Edward Cooper, will preside. Few hospitals in London of equal standing are less in the public eye; yet it was the first of its kind in London, and was established in 1801. It possesses laboratories, but not an adequate nurses' home, to which £5,000 of the £55,000 asked for will be devoted. The secretary, Major W. J. H. Brand, died recently, after launching the special appeal.

### AEROPLANE RESEARCH.

The letter from Sir Ronald Ross appearing in the *Times* of February 7 under the heading of "Medical Service by Air" opens a number of interesting speculations as to the possibility of "medical exploration of Africa and other medically dark countries." The writer explains himself as unacquainted with the aeronautical feasibilities, and in this limitation we ourselves must join him; but in his suggestion there is much more than the casual reader may at first sight discern. In peace, and particularly in war, the mobile medical unit has already established itself, and, of still more recent origin, we have the mobile pathological laboratory. In the space available on modern aircraft some such adaptation could almost certainly be made. Typical of the writer, the letter gives a suggestion well worth consideration to the inventors and organisers of such expeditions, and, as he tells us the matter has already received a certain amount of discussion, we await the next chapter with interest.

### TESTING OF SCIENTIFIC GLASSWARE.

So many branches of medical work bring one into contact with one or other of the graduated glassware instruments that our readers will not fail to appreciate the importance of the new facilities recently announced by the authorities of the National Physical Laboratory, Teddington, with regard to the standardisation of laboratory glassware. Relatively little attention was given in this country to volumetric glassware as a manufacturing industry in pre-war days, and now the work has so satisfactorily developed, the position gained must never be lost. Apart from mere production, the most essential and vital point is calibration, and in providing ready possibilities for the British maker to maintain a high standard of excellence in this direction the Teddington authorities, who undertake standardisation work for extremely modest charges, are providing most valuable national assistance.

### TEACHING DEVELOPMENTS.

Coming with the present wave of medical teaching reform, which has fortunately so far taken place along the lines suggested by the Royal Commission on University Education in London and strongly advocated by Sir George Newman in his memorandum, we note that the governing body of the London School of Tropical Medicine, taking advantage of the opportunities coincident with the removal of their school to Euston Square, are bringing their whole-time appointments into line with the professional units and "elements" schemes. Whole-time directorships have been instituted in protozoology, helminthology, entomology, and tropical pathology, and others will be added later. Improved post-graduate education in these particular subjects is of the most urgent necessity, and we join with the *B.M.J.* in the hope that it will receive that financial support which it deserves from the University Grants Committee now considering the needs and claims of the various educational bodies in this country. The appointments to other new clinical teaching "elements" may be supplemented by the names of two distinguished physicians, who have been appointed director and assistant director of the unit at St. Bartholomew's Hospital. These are Sir A. E. Garrod, K.C.M.G., and Colonel Francis Fraser. Both of these gentlemen have a distinguished war service to their credit, and are exceedingly well known in the world of medical research.

### MEDICAL BENEFITS.

We read that a letter has been forwarded by the Federation of Medical and Allied Societies to all Members of Parliament begging them to support the demand for an inquiry into the regulations under which medical benefit is obtained through panel practice, before any expansion or alteration in the provisions of the Insurance Act is proposed. This action arises as a result of the reply given by Dr. Addison to the deputation which he received a few days ago. He then promised that he would unhesitatingly recommend that such an inquiry be made after receiving a report from the Medical Consultative Council, should he thereby become convinced that advantage was to be derived. The Council must speak first, and the Federation will then be in a stronger position to criticise any existent or intended methods, and, meanwhile, they wish to keep before Parliament the urgency of the whole question involved.

### THE LIMITED LIST.

The new arrangements which were recently adopted by the Croydon Insurance Committee for the local administration of medical benefit, and which formally come into operation on April 1 this year, include:—(1) Limitation of a practitioner's list to 2,000 insured persons; (2) an insured person is given a more frequent right to change his doctor (twice a year, instead of annually); (3) the medical card is accepted as evidence of the person's right to medical benefit; (4) the practitioner has no right to receive fees for the treatment to any insured person otherwise than for treatment outside his agreement. The practitioner must advise the patient as to the method of, and assist him in, obtaining any special treatment he may require. In adopting these changes the opinion was expressed that the new regulations would secure an improvement in administrative machinery, a better understanding of the steps to be taken by insured persons, and a clearer definition of the obligation of practitioners and chemists.

### A DIPLOMA IN TUBERCULOSIS.

The knighthood recently conferred upon Sir Henry Gauvain, medical superintendent of Lord Mayor Treloar Cripples' Hospital and College, Alton, Hants, has been followed by a dinner in his honour, presided over by the founder. In support of the toast Viscount Burnham said that tuberculosis had become a new danger since the war, since emigrants from the underfed peoples of Central Europe would probably carry the disease into the countries of their adoption. At Alton and at Hayling Island it was possible for Sir Henry Gauvain to pursue research while engaged in treatment, and, we add, Alton is a useful centre also for students of pulmonary consumption to attend because they can add to their knowledge by study of the surgical aspect of the subject. In reply, Sir Henry Gauvain said that the hospital had accomplished pioneer work. It had begun with twenty, and now 350 patients were treated in the institutions. He wanted to see 1,000 patients in them. The post-graduate work pursued at Alton needed one encouragement—namely, a diploma in pulmonary and surgical tuberculosis at one of the great universities. Sir Norman Moore, P.R.C.P., said that the hospital and its staff had performed a national service. The part played by Mr. J. Hall Richardson in the inception of the idea was also referred to; and we hope the dinner in honouring Sir Henry Gauvain will have enlightened the public still further in the work and aims of one of the most interesting of hospitals.

### THE FIRST ANNUAL REPORT.

The first annual report of a voluntary hospital reached us before the close of January. It is of the West Suffolk General Hospital, Bury St. Edmunds, and is quite a business-like publication, with the statement of "Receipts and Disbursements" made up to December 31, 1919, and duly audited. Complete information as to the numbers of patients (the hospital is one of 100 beds), medical classification, lists of investments and subscribers, etc., is included. The accounts are not prepared on the uniform plan now adopted almost everywhere, but, apart from this, we have nothing but praise to offer in respect of this prompt publication. We are looking forward to the first annual report prepared on orthodox lines.

### A NEW CHILDREN'S HOSPITAL READY.

Before he died in August 1914, the late Mr. John Sykes, of Lindley, Yorkshire, appointed trustees under his will to build a hospital for adults and children, to be known as Bradley Gate Sanatorium. He presented a site and pavilion to the town for the treatment of surgical tuberculosis. The trustees have informed the town council that the part of the sanatorium intended for children is complete, and ready to be presented to the town council. The pavilion will accommodate twenty-four beds, twelve for boys and twelve for girls. Between the two general wards is a large day-room where games for both sexes can be played. The pavilion, which is an institutional unit of several departments, is designed for open-air treatment with an open balcony along its whole length. The equipment is also installed. One of the people consulted by the late Mr. Sykes was Sir Henry Gauvain, medical superintendent of Alton Cripples' Hospital The corporation was consulted about the site, which comprises thirteen acres. Mr. Sykes bought it from Mr. Blamires and conveyed it to the corporation. The cost, amounting to £12,000, was paid out of the estate. During the war the buildings were used as an auxiliary military hospital. A year ago the War Office gave possession to the trustees, and since then the architect, Colonel Cooper, has been busy completing the pavilion.

### A COMPARISON BETWEEN BIRMINGHAM AND LEEDS: THE CO-ORDINATION MOVEMENT.

Though there is little to add at the moment to the scheme already published in The Hospital for the relief of the waiting-lists at the Birmingham voluntary hospitals by the admission of their cases into the guardians' infirmaries at Selly Oak and Dudley Road, progress can be reported. Shortly before the end of January the Birmingham Board of Guardians adopted the special report of its infirmaries' committee, and this report shows that a good beginning has been made. The chief medical officer is debating the details; an informal conference between the guardians and the voluntary hospital committees, the University, and the Health Committee has been held; and a joint committee has sprung from it. The plan is, virtually, to restrict admission to those in need who are resident within the area of the union, to extend the infirmaries' special departments and to add to them, and to notify the public of the change by a change of name, so that in future the institutions will be known as the Dudley Road and Selly Oak hospitals, even if their place names are not altered as well. From every quarter the co-ordination of our hospital system is being completed, and each local scheme illustrates some omission in another. Thus the statesmanlike plan now started at Leeds, which we gave in detail last week on page 437, co-ordinates the public and the hospitals, but omits from its immediate purview the guardians' hospitals. Though the latter in Leeds have plans of their own, those plans should be related to the income-raising policy. Similarly, Birmingham, which is ahead in co-ordinating the voluntary and the municipal hospital services, should take a hint from Leeds and start, on the lines we report, an employers' contribution fund.

### THE CONVERSION OF AN AERODROME: A DOCTOR'S FAITH.

We lately alluded to the desperate finances of the West Kent General Hospital, Maidstone, and there goes with this, as always, a serious shortage of beds. In Marsham Street, where the hospital lives, extension would be difficult. How then can it be carried out, with neither funds nor land? An ingenious solution is proposed by Dr. H. Clifford Barclay, of Maidstone, who lately paid a visit to Detling, which made a great impression on his mind. At Detling there is an aerodrome, which he inspected, and an empty block of buildings allied thereto. Dr. Barclay therefore suggests that the committee of the Maidstone Hospital should ask the Government not to sell the buildings for a song, but to let them at a peppercorn rent, so long as they are used as an addition to the hospital. Dr. Barclay is convinced that the buildings are suitable, and need only decoration, while the men's huts could be used, he says, "to make corridors for the staff." He enlarges on the value of the site, not only for open-air wards, but in relation to food, which will be "obtainable almost at the door," and says that the site offers an excellent position for a hospital garden. We hope that the committee of Maidstone Hospital will consider this idea fairly and thoroughly, if only because, were it practical, it would appeal immensely to the local public, and therefore attract support which no other extension scheme less immediate in effect and less romantic in origin could do. Is it a good idea? We ask because too often the better an idea is the less are ordinary folk inclined to study it fairly.

### WOMEN'S DEBT TO THE HOSPITALS.

The Rev. G. B. Cronshaw, treasurer of the Radcliffe Infirmary, Oxford, generally has something of value to say when he presents his annual statement. This year he made a good point in his appeal for probationers, of whom the Radcliffe, like other hospitals, is very short. The efficiency of a hospital, he said, is the efficiency of its nurses, and if the authorities do all they can to make the nurse's life a full and happy one, then women with a latent vocation for the work must come forward in the interests of their weaker brethren. There is force in this argument, which appeals alike to the head and to the heart, and it is well to hear that when the hospitals open a fit career for intelligent women the women also owe a duty to those who have provided a new opportunity for their usefulness.

### THE CHURCH'S PART IN THE SUNDAY FUND.

Since the support of hospitals began with those infirmaries attached to monasteries and religious houses, and was definitely associated with the national religion, it is interesting to record the continuity of that process in the present religious life of the nation, and especially with the Establishment. In the monthly organ of the Central Church Defence League, the *National Church*, there is an analysis of the contributions of the different places of worship to the total of the Hospital Sunday Fund, which amounted in 1919 to over £40,000, a total, however, nearly £3,000 less than was raised by the Fund in 1918. To this, the newspaper records, the contribution of the Church of England was £29,390. The synagogues came second with a total of £2,053; the Congregationalists, Wesleyans, Baptists and Presbyterians supplied severally over £1,000. Next came the Roman Catholics with £938, and the last of the specified denominations is the Greek Church, which contributed £121. The Church of Scotland raised £593. Does this reflect the influence of the several bodies in the religious life of London?

### HOSPITALS AND HOUSING SCHEMES.

Another difficulty of the building problem besets hospitals at present. Many houses have been ordered. Some contracts are eight months old, and yet in many places not one of the specified houses has been finished. The reasons are a lack of material and an even more serious lack of transport. In these circumstances a proposal to build a new hospital may be held to compete and interfere with a local housing scheme. When the proposal comes from a board of guardians or local authority responsible or allied to the authority responsible for the new houses, the proposers are in a quandary. It has been felt at Derby, where only after prolonged debate has the board of guardians decided to seek the sanction of the Ministry of Health to build a new infirmary. Perhaps one of the advantages of the voluntary system is that it allows a hospital committee to seek the best for its patients, unharassed by the considerations of policy and vote-catching which no municipal body can avoid.

### ACCOUNTS AT EXPRESS SPEED.

Some hospitals take a commendable pride in the promptitude with which their annual accounts are passed for audit, and in the more difficult feat of obtaining a refund of income tax with equal speed. We do not know what records have been established in this connection, but the Warneford, Leamington and South Warwickshire Hospital was early in the field last month. The balance-sheet and all subsidiary accounts for the year ending December 31, 1919 were ready for the auditor's signature on January 15, 1920. Two days later, that is to say, on January 17, the cheque for the refund of income tax was received from the Inland Revenue. If the accounts themselves balanced as easily as they were drafted and audited, the hospital would have little to complain of, but alas, there was an adverse balance of £1,176 on a total of £1,401. We suppose that no hospital can shine at all points, and Mr. C. R. W. Offen, the house governor, would probably admit that some of the blame for this must be divided with the subscribers.

### MR. LARKING TELLS THE TRUTH.

The Norfolk and Norwich Hospital has an accumulated deficit of about £20,000. The Special Appeal Fund for an orthopædic department and other extensions now reaches £21,000. Among the donations was one of £100 from the King. The Board, in its quarterly report, emphasised the need for the reorganisation of the outpatient department, and the building of a Maids' Home, and improving heating and hot-water services. Mr. Charles Larking, the chairman of the Finance Committee, said the total sum for the orthopædic department and the new heating scheme would be about £60,000. The public threw £40,000 of work upon the hospital in a year and only supplied £25,000. Mr. Larking severely criticised the parsimonious treatment of the Hospital by the big firms. "The prosperous business firms are those who can help the Hospital most. The employees do their part, the county people do theirs, and so do the friendly societies. But the big firms are not doing what they ought to support the Hospital. It is not fair for a firm employing hundreds of hands to subscribe from £2 2s. to £5 5s. a year when the cost of one letter of recommendation is £8 13s. 6d. It is scandalous that such a state of things should exist when it is well known that if the Hospital were put on the rates it would mean 1s. to 1s. 6d. in the £ to the ratepayers, and all the large firms would have to pay." We are glad to see this frank criticism. The remedy elsewhere has been to form an organisation for the big firms and to get them to agree to the *pro rata* subscriptions in proportion to the number of their employees. The report was adopted and the Lord Mayor (Mr. George Green), who presided, declared that he would do his best to take the question up and strive to make a city appeal.

### THE DREADNOUGHT ON THE DOWNS.

The opening of the new hospital for Tropical Diseases, that branch of the Dreadnought Seamen's Hospital which has been moved to Endsleigh Gardens, is only the first of two plans which the Seamen's Hospital Society is putting on foot. The second is for a sanatorium for seamen suffering from consumption. When they do fall victims it is often difficult to place them in a sanatorium because one of the disadvantages of a seaman's life is that the sea is not a domicile, though it may be a home or a grave; and with the demands for sanatorium treatment each institution is kept busy to find a colourable reason for regarding some unfortunate applicant as ineligible unless he can prove his domicile. A site, therefore, for a seamen's sanatorium is wanted. It is hoped to place it on the Hampshire Downs. The ease with which the funds are raised will be an index to liveliness of the public memory. Nothing should be too much for our seamen. It is now said that the war was won by the blockade. Who enforced that? Who sacrificed their lives in silence; whose work was more effective than theirs? If a seaman is neither seen or heard, he should be rewarded for this unobtrusiveness by receiving from the public the sanatorium which he needs in tuberculosis.

### THIS WEEK'S DRUG MARKET.

The features of the market are much the same this week as they have been for some weeks past—namely, a rather quiet demand at home and a very active one from abroad, and a steady upward tendency of prices. Values of the synthetic drugs are firmly maintained, several of them, notably resorcin and hexamine, being particularly scarce. Supplies of bromides continue to come from Germany, but not in sufficient quantities to bring the price down. Santonin is again dearer. The rise in value of this drug must be a record, even in these days of big prices, since the present value is 120 times the figure at which it could be bought at one time. Tartaric acid and citric acid are in strong demand, and prices are higher. Castor oil is dearer. Menthol and camphor are unchanged. Cocaine seems likely to be still dearer.

